# Composition and Functional Specialists of the Gut Microbiota of Frogs Reflect Habitat Differences and Agricultural Activity

**DOI:** 10.3389/fmicb.2017.02670

**Published:** 2018-01-11

**Authors:** Bing-Hong Huang, Chun-Wen Chang, Chih-Wei Huang, Jian Gao, Pei-Chun Liao

**Affiliations:** ^1^Department of Life Science, National Taiwan Normal University, Taipei, Taiwan; ^2^Technical Service Division, Taiwan Forestry Research Institute, Taipei, Taiwan; ^3^The Key Laboratory for Silviculture and Conservation of Ministry of Education, College of Forestry, Beijing Forestry University, Beijing, China; ^4^Faculty of Resources and Environment, Baotou Teachers’ College, Inner Mongolia University of Science and Technology, Baotou, China

**Keywords:** 16S rRNA metagenome, adult Anura, functional predictions, gut microbiota, agricultural activity

## Abstract

The physiological impact of agricultural pollution, habitat disturbance, and food source variability on amphibian remains poorly understood. By comparing the composition and predicted functions of gut microbiota of two frog species from forest and farmland, we quantified the effects of the exogenous environment and endogenous filters on gut microbiota and the corresponding functions. However, compositional differences of the gut microbiota between the frog species were not detected, even when removing roughly 80–88% of the confounding effect produced by common and shared bacteria (i.e., generalists) and those taxa deemed too rare. The habitat effect accounted for 14.1% of the compositional difference of gut microbial specialists, but host and host × habitat effects were not significant. Similar trends of a significant habitat effect, at an even higher level (26.0%), for the physiological and metabolic functions of gut microbiota was predicted. A very obvious skewing of the relative abundance of functional groups toward farmland habitats reflects the highly diverse bacterial functions of farmland frogs, in particular related to pathogenic disease and pesticide degradation, which may be indication of poor adaptation or strong selective pressure against disease. These patterns reflect the impacts of agricultural activities on frogs and how such stresses may be applied in an unequal manner for different frog species.

## Introduction

Host habitat is the primary filter of the gut microbial community (Ley et al., 2006; Sullam et al., 2012). Through food intake, the gut becomes a reservoir of microbiota originating from the external habitat (Drake and Horn, 2007; Wiggins et al., 2011). For example, amphibians acquire soil microorganisms through the ingestion of prey and their own shed skin, both of which are covered with soil bacteria (Wiggins et al., 2011). The gastrointestinal environment also acts as a second-layer filter for selecting microbes that arrive from the external environment (Feld et al., 2008; Thomas et al., 2011). Epithelial cells and the fluids of the digestive tract maintain a homeostatic environment (Artis, 2008; Kohl et al., 2013) providing a constant adaptive pressure on intestinal microbes. These host effects which may potentially affect gut microbial composition are called endogenous factors. Gastrointestinal microbial assemblages also reflect the dispersal processes of hosts (via habitat shifts), environmental selection, and ecological drift (Costello et al., 2012). These effects are often called exogenous factors. These exogenous and endogenous factors could synergistically shape the gut microbial community. For example, the tolerance, interaction, and adaptation to a specific niches, which is so called host adaptability, could also alter gut microbial composition (Hooper et al., 2002; Spor et al., 2011). Gut microorganisms reflect evolutionary selection pressure acting via the adaptation of the host to the external environment. The habitat-selected host genotypes may filter out immigration of unsuitable microorganisms, and may facilitate first colonization of mutualistic or pathogenic microbes from co-existence neighbors or parents (e.g., Lawley et al., 2008). The host adaptability to the habitat, the host internal (gut) environment, and dynamic of external and internal bacteria could together shape the gut microbial community (Ley et al., 2006).

The influence of habitat change on the homeostasis of gut microbiota is of particular importance for understanding the adaptability of hosts that undergo changes to their niche (Spor et al., 2011). Habitat-specific gut microbiota demonstrate how the external environment mediates the intestinal environment (Ley et al., 2006; Sullam et al., 2012; Wong and Rawls, 2012; Giatsis et al., 2015; Bletz et al., 2016; Chang et al., 2016). The functional convergence of differing gut microbial assemblages under shifting habitats indicates the taxonomic incoherence and metagenomic plasticity of gut microbiota (Bletz et al., 2016). Studies of primates have shown that artificial disturbance and habitat degradation decrease gut microbial diversity (Amato et al., 2013; Barelli et al., 2015), and can affect hosts’ metabolism and health (Amato et al., 2013). Numerous medical studies demonstrate the association between gut microbiota and hosts’ disease (Artis, 2008; Feld et al., 2008; Barbosa and Rescigno, 2010; Garrett et al., 2010; Manichanh et al., 2010; Schwabe and Jobin, 2013; Bultman, 2014; Hullar et al., 2014; Jasarevic et al., 2015; Johnson et al., 2016), highlighting the relationship between gut microbiota and the adaptability of a host. However, such relationships between gut microbiota and host physiology have focused primarily on experimental animals or humans. Few studies have included fauna in a natural setting.

Amphibians are highly sensitive to the environment and are very suitable for studying adaptability (Hopkins, 2007), although some have suggested caution (Kerby et al., 2010). Through the functional prediction of the gut microbiota, we may be able to more clearly understand the physiological status and niche divergence of species under differing environmental conditions (Stevenson et al., 2014; Bolanos et al., 2016). Furthermore, the functional change of the gut microbiota may be even more sensitive to impacts of environmental disturbance than the hosts themselves (Amato et al., 2013; Barelli et al., 2015).

Vertical transmission of maternal microbes through birth (and breastfeeding in mammals) also modifies the composition of gut microbiota assemblages (Funkhouser and Bordenstein, 2013), which also indirectly affects neurodevelopment (Jasarevic et al., 2015). However, it is hard to detangle the relative effect of external environment and vertical transmission on gut microbial community. Adult frogs offer this possibility. Significant differences between the gut microbiota of tadpoles and adult frogs have been demonstrated (Kohl et al., 2013), and change of the diet is the main contribution for the turnover of gut microbial composition during metamorphosis and distinct gut microbial composition among habitats (Vences et al., 2016). In tadpoles, as with fishes, there is a higher abundance of operational taxonomic units (OTUs) from the phylum Proteobacteria while Firmicutes and Bacteroidetes, common to terrestrial amniotes, dominate the gut environment in adult frogs (Kohl et al., 2013). The change of dietary strategy (from aquatic herbivore to a typically terrestrial insectivore) and gut pH, as well as the development of a gastric stomach and an epithelial immune function through metamorphosis of anuran amphibians (Hourdry et al., 1996; Du Pasquier et al., 2000) combine to reset the gut environment and succession of intestinal microflora (Vences et al., 2016), thereby eliminating influence from vertical transmission in mature frogs.

We selected two phylogenetically distinct frog species, *Fejervarya limnocharis* and *Babina adenopleura*, as hosts for the assessment of gut microbial composition. Both species are on the IUCN Red List Least Concern faunal list. *F. limnocharis* is usually found near the paddy fields, ponds, lakes, and ditches, while *B. adenopleura* is usually found in ponds or swamps (Supplementary Table S1). Both species have broad diets. Similar prey for both species includes Arachnida, Coleoptera, Hemiptera, Hymenoptera, Isoptera, Lepidoptera, Orthoptera, and Stylommatophora. *F. limnocharis* has a more diverse diet than *B. adenopleura*, also feeding upon Blattodea, Collembola, Dermaptera, Diptera, and Psocoptera (Supplementary Table S2). Both species may be found together in similar habitats. We chose a forest habitat and a farmland habitat. These two sampling sites have an altitudinal difference of ∼340 m (F: ∼60 m a.s.l.; N: ∼400 m a.s.l.) and are situated ∼3 km away from each other, separated by the Beishi and Nanshi rivers, upstream of their confluence with the Xindian River. Traveling between these two sites seems to be infrequent because long distance migration is often maladaptive to juvenile frogs (Smith and Green, 2005; Semlitsch, 2008).

In this study, we compare 12 gut microbial communities, three samples for each of four different combinations (*F. limnocharis*–forest; *F. limnocharis*–farmland; *B. adenopleura*–forest; *B. adenopleura*–farmland). The four combinations were set and have passive interactions with each other (e.g., same species with different habitat or same habitat with different species). We determine whether the internal or external environment of a host, that is the host itself or its habitat, most strongly determines the compositional and functional diversity of the gut microbiota. Three main issues questions arise from this approach: (1) to what degree do external and internal environments filter the gut microbiome? (2) How different is the composition of gut microbial assemblages within sympatric hosts, and how similar is the gut microbial composition between allopatric host populations of the same species? (3) Do the predicted functional groups of gut microbiota reflect the characteristics of hosts and habitats? For testing the role of host and habitat effect on the composition of gut microbiota, we sampled two frog species from both paddy fields and ponds in a forest setting. We determined the relative abundance (RA) of gut microbiota using high-throughput 16S rRNA gene sequencing of each individual frog. We defined a gut microbial metacommunity as a gut microbial community that had either the same host species or the same habitat. As such, we could compare the microbial community compositions between different hosts and between different habitats. Since the common microbes and those deemed as “too rare” could confound the results, we removed these microbes and retained only the host and habitat specialists for quantifying the host and habitat effects on gut microbiota composition. In addition, *in silico* profiling predicted and classified the metabolic and physiological functions of these gut microbiota. To elucidate the impact of the anthropogenic (agricultural activity) interference on the ecological functions of gut microbiota, we quantified the functional divergence between the hosts and habitats.

## Materials and Methods

### Ethics

We sacrificed 12 frogs for obtaining the intestinal microbiota. To prevent contamination from bacteria outside the sample, the forceps and scissors for obtaining the intestinal tissue were sterilized by both autoclave and UV-light. The tissues were stored in -80°C before extraction. The Institutional Animal Care and Use Committee, National Taiwan Normal University (No. 104033) reviewed and approved the study protocols and the number of animals that could be used. All experiments involving animals followed the principles of the 3Rs (replace, reduce, and refine) to prevent excessive and unnecessary killing.

### Sampling Sites

For this study, two sites near Taipei, Taiwan, having different environmental conditions, were sampled for *F. limnocharis* and *B. adenopleura*. The natural habitat (N) site is a secondary growth forest near a forest road and is adjacent to some small ponds (24°53′N, 121°33′E). The farmland (F) site lies in an agricultural field that produces rice and vegetables (24°55′N, 121°32′E).

### Metagenomic Experiments

Intestinal microbial metagenomic DNA was extracted following the protocol of Sharma et al. (2003). Every extracted metagenomics DNA were adjusted to 50 ng/μL for subsequent analysis. We amplified the V4 hypervariable 16S rRNA region using the primers 27F (5′-AGAGTTTGATCCTGGCTCAG-3′) and 533R (5′-TTACCGCGGCTGCTGGCAC-3′). Double distilled water was used as a control to ensure no contamination during amplification. The DNA library was assembled using a Roche GS FLX Titanium emPCR kit (Roche Applied Science, Indianapolis, IN, United States). We then sent the DNA libraries to Welgene Biotech Co., Ltd. (Taipei, Taiwan) for pyrosequencing. A Roche 454 FLX Titanium instrument and reagents were used for pyrosequencing and procedures followed the manufacturer’s instructions. We removed V4 sequence fragments that were shorter than 200 bp, or that had barcodes, polyN, or polyA/T. We also eliminated readings having a quality score <Q25. The trimmed sequences were analyzed and aligned using the software mothur (Schloss et al., 2009). We normalized each OTU by its copy number using normalize.shared function implemented in mothur. We considered sequences that were >97% identical to be the same species and thus represented an OTU. Each OTU was classified using the SILVA rRNA database. We removed chimeras using the UCHIME algorithm (Edgar et al., 2011). The raw sequence data were deposited into the NCBI GenBank under Bioproject PRJNA279212 (accession number: SAMN04158746 for *B. adenopleura* and SAMN03434989 for *F. limnocharis*).

### Microbial Community Diversity

We performed a rarefaction analysis to estimate the probable richness of each microbial community sample. To reduce the effect of sampling effort, we drew species accumulation curves using vegan packages of R (Dixon and Palmer, 2003) to correlate the number of microbial taxa and the number of sampled frogs and thereby assess the taxa-abundance distributions (van der Gast et al., 2011, 2013).

### Functional Predictions of the Gut Microbiomes

For predicting physiological and metabolic functions of gut microbiota, we used PICRUSt v. 1.0.0, a functional prediction tool for estimating the shared gene content according to the corresponding microbiome phylogeny (Langille et al., 2013). PICRUSt can use an extended ancestral-state reconstruction algorithm to generate the composition of gene families for each metagenome. We used the online version of PICRUSt—Galaxy (https://huttenhower.sph.harvard.edu/galaxy/)—for assisting with our algorithms. In the PICRUSt prediction, the Greengenes v. 13.5 OTUs database (DeSantis et al., 2006) assigned the cleaned sequences to a closed reference OTU table using the 97% similarity implemented in QIIME v. 1.8.0 (Caporaso et al., 2010). We reconstructed and predicted the functional contribution of each OTU member by mapping 16S sequences with their nearest reference genome. A “virtual” metagenome with gene content abundance was then generated using the Kyoto Encyclopedia of Genes and Genomes (KEGG) Ortholog. The abundance of each KEGG Orthologs category was presented in a KEGG pathway at the third hierarchical level.

### Comparing the Compositional Distribution of Gut Microbes from Different Hosts and Habitats

We used Venn diagrams to compare the intersections and unions of gut bacteria and functional groups among the different metacommunities. We used the ANOSIM function in the vegan package in R, which calculates difference of mean ranks between (*r*_B_) and within (*r*_W_) metacommunities (Clarke, 1993) to assess the dissimilarities of microbial composition between hosts and between habitats. We also used ggtern package in R to draw Ternary diagrams (Hamilton, 2016) to assesse the differences in the abundance of bacterial compositions or functional groups between hosts or habitats. For the ternary diagrams, we used the bacterial composition (or functional groups) of the other host as a baseline for comparing the compositional differences (or differences of functional groups) of the target host between habitats. For example, the bacterial composition of *B. adenopleura* was used as the benchmark to compare the compositional differences of gut bacteria of *F. limnocharis* between forest and farmland. Similarly, we used the bacterial composition (or the functional groups) of one habitat as the benchmark for comparing the gut bacterial composition or functional groups between different hosts in the other habitat.

### Data Filtering for Identifying the Specialists

Because “everything is everywhere” (Baas-Becking, 1934) and our purpose was to find the environment that selects and those taxa that are selected, we first wanted to remove those species not selected (i.e., the generalists) and identify those potentially being selected (i.e., the specialists). We used the supermajority rule (2/3 RA) to classify the generalist and specialist microbes of host habitats and host species (Chazdon et al., 2011) using the function CLAM in the R package vegan. We discarded those OTUs classified as “too rare.” Similarly, we retained the functional group specialists of the host and habitats for the further analyses.

### PCA and PERMANOVA

We performed principal component analysis (PCA) for two reasons: (1) to access the clustering pattern by hosts and habitats, and (2) the axis of PCA can provide quantitative weight on our variables, and can be used to transform the compositional matrix into vectors following the explanatory proportion for further analysis of multivariate logistic regression instead of principal coordinate analysis which used non-Euclidean distance matrix (Ramette, 2007). We performed PCA using the R package factoextra (Kassambara, 2015). As well, permutational multivariate analysis of variance (PERMANOVA) estimated the significance of the variance and covariance of independent factors “host habitat” and “host species” on the first three PCs for microbial composition and predicted functional groups (53.02 and 75.08% variation, respectively) using 999 permutations in the R package vegan (Dixon, 2003).

### Redundancy Analysis to Assess the Explanatory Proportion of the Host and Habitat Effect

For understanding how host species and habitats affect the RA of gut microbiota, we applied distance-based redundancy analysis (dbRDA) to estimate the explanatory proportion of the RA of microbial compositions and functions. Analysis of variance (ANOVA) tested the significance of each independent factor through 999 permutations under a reduced model using the capscales function in the R package vegan.

## Results

### High Beta-Diversity of Gut Microbial Communities and the Underestimation of Gut Bacterial Richness

We sequenced a total of 232,153 reads, retaining 197,260 reads (mean of 16438.33 reads per sample, range 7346–33,441 reads) for analyses after discarding (cleaning) the substandard sequences. Among these cleaned sequences, we obtained 562.33 ± 198.07 OTUs per sample (range 291–1011 OTUs) using the 97% similarity criterion for determining OTU (**Table 1**). The sequence depth obtained a mean richness of 76.87% (54.48–85.66%) or 77.06% (47.15–87.35%) as estimated through the Cha01 or ACE indices, respectively (**Table 1**). This suggested an underestimation of gut bacterial diversity in our sampling. This underestimation was also revealed by the linear increase of bacterial OTUs in the microbial assemblages of individual samples (Supplementary Figures S1–S3).

**Table 1:** Summary statistics of the 16S rRNA metagenome sequencing.

Host species	Habitat	Sample	Reads for cleaning			OTUs	ACE (95% CI)	Cha01 (95% CI)	Shannon (95% CI)	Simpson (95% CI)
			1st	2nd	Final					
*F. limnocharis*	Forest	AN1	17,442	13,700	12,380	540	640.45 (609.75-684.66)	653.55 (611.58-720.15)	4.878 (4.850-4.907)	0.024 (0.022-0.025)
*F. limnocharis*	Forest	AN2	17,343	15,656	14,737	291	361.32 (334.88-403.68)	370.88 (334.72-436.92)	3.552 (3.522-3.583)	0.088 (0.085-0.091)
*F. limnocharis*	Forest	AN3	37,167	34,860	33,441	458	524.32 (501.48-559.15)	541.72 (507.34-600.06)	3.420 (3.398-3.442)	0.102 (0.100-0.104)
*F. limnocharis*	Farmland	AF1	18,438	16,714	15,906	660	797.25 (759.73-848.88)	794.10 (749.07-861.90)	4.975 (4.950-5.001)	0.018 (0.018-0.019)
*F. limnocharis*	Farmland	AF2	23,334	21,971	20,716	1011	1358.82 (1287.07-1449.22)	1385.21 (1289.75-1513.36)	5.297 (5.275-5.320)	0.013 (0.013-0.014)
*F. limnocharis*	Farmland	AF3	20,095	18,963	18,403	590	863.05 (797.64-949.06)	839.50 (766.51-942.68)	2.960 (2.928-2.993)	0.152 (0.148-0.155)
*B. adenopleura*	Forest	BN1	15,562	13,495	12,137	473	577.71 (544.93-625.44)	573.44 (535.28-635.01)	4.760 (4.734-4.786)	0.017 (0.017-0.018)
*B. adenopleura*	Forest	BN2	17,135	15,531	14,281	523	611.29 (583.42-652.02)	610.55 (576.78-665.50)	4.881 (4.857-4.905)	0.015 (0.015-0.016)
*B. adenopleura*	Forest	BN3	11,948	8806	7346	427	511.07 (483.76-551.52)	512.85 (479.00-568.75)	4.700 (4.663-4.736)	0.024 (0.022-0.025)
*B. adenopleura*	Farmland	BF1	17,583	16,639	15,983	864	1104.41 (1051.07-1172.96)	1146.40 (1069.16-1252.73)	4.465 (4.430-4.499)	0.050 (0.048-0.052)
*B. adenopleura*	Farmland	BF2	17,350	16,103	15,564	583	825.47 (765.75-904.72)	842.37 (763.24-956.24)	3.568 (3.534-3.603)	0.097 (0.094-0.100)
*B. adenopleura*	Farmland	BF3	18,756	16,578	16,366	328	695.60 (619.92-790.92)	602.04 (506.14-749.57)	1.356 (1.322-1.390)	0.583 (0.573-0.592)

*OTUs, operational taxonomic units defined by 97% identity threshold of the 16S rRNA gene; ACE, abundance-based coverage estimator.*

### Major Bacterial Groups Dominate Gut Microbial Communities Influenced by Habitat

**FIGURE 1:**
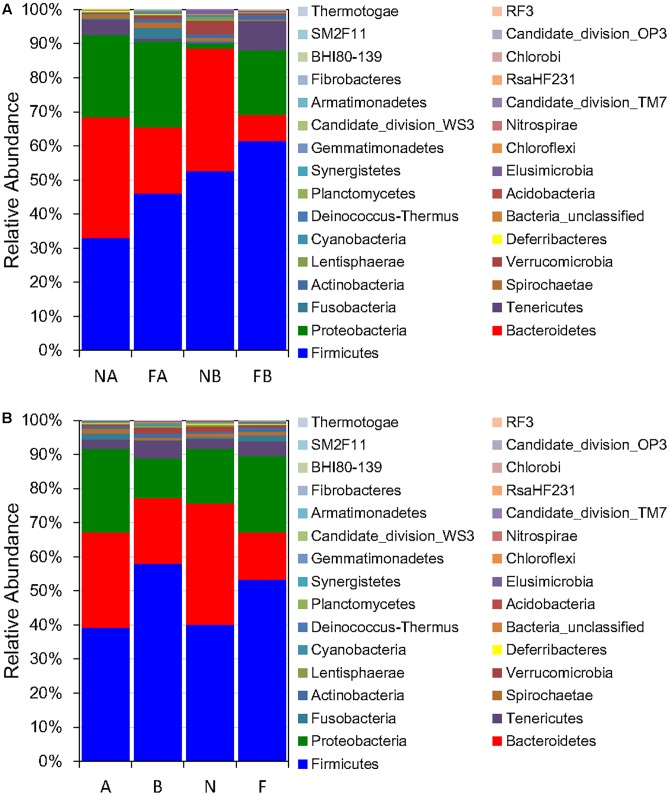
Relative abundance of microbial phyla **(A)** classified by both habitat and host (F, farmland; N, forest; A, *Fejervarya limnocharis*; B, *Babina adenopleura*, *n* = 3 each), and **(B)** classified by either habitat or host (*n* = 6 each).

The five most dominant (top 5) phyla of bacteria were Firmicutes, Bacteroidetes, Proteobacteria, Tenericutes, and Verrucomicrobia. These phyla accounted for >90% of the gut microbial community composition for both frog species in both habitats (97.25 and 94.55%, for *F. limnocharis* and *B. adenopleura*, respectively, in the forest samples and 92.37 and 96.83% for *F. limnocharis* and *B. adenopleura*, respectively, in the farmland samples; **Figure 1** and **Table 2**). These values are roughly consistent with previous studies showing that these microbial phyla dominate amphibian gastrointestines (Kohl et al., 2013; Colombo et al., 2015; Vences et al., 2016; Weng et al., 2016; Zhang et al., 2016) However, RA differed slightly between sample habitats, in particular for the phyla Bacteroidetes and Proteobacteria (Mann–Whitney test and Kruskal–Wallis test, *P* < 0.05; **Table 2**). This significant difference for RA between habitats may reflect the frogs having different life habits, such as diet, for the different environments (Chang et al., 2016). However, as we know that both frog species have certain divergent feeding strategies (Supplementary Table S2), we wondered if there was an influence of the interaction between host and habitat on these dominant gut bacterial phyla. Hence, we performed a two-way ANOVA and revealed that only Bacteroidetes, a microbial phylum functionally involved in polysaccharide degradation (Thomas et al., 2011), had significant differences of variance between habitats (*P* = 0.022). Frog in two habitat types also revealed difference in food resources (Supplementary Tables S1, S2; Chang et al., 2016). We detected no significant effects for other phyla nor for the host effect nor for habitat × host (**Table 2**). This implied that only the habitat mattered and that not all bacterial groups were affected equally. This assessment supports the hypothesis of a constant gastric environment for maintaining an invariable core of microbiota. Only certain microbial indicators reflected the disturbance of the external environment (Barbosa and Rescigno, 2010; Garrett et al., 2010).

**Table 2:** Relative abundance of the five most dominant” microbial phyla in the gut of sampled frogs and the significance tests for the differences of relative abundance between host species and between host habitats.

Phylum	Relative abundance				MW test^a^		KW test^b^		Two-way ANOVA^c^		
	AN	AF	BN	BF	Host	Habitat	Host	Habitat	Host	Habitat	Host × habitat
Firmicutes	0.377 ± 0.105	0.459 ± 0.016	0.566 ± 0.175	0.611 ± 0.215	0.180	0.699	0.150	0.631	0.143	0.562	0.869
Bacteroidetes	0.410 ± 0.138	0.195 ± 0.099	0.324 ± 0.146	0.076 ± 0.053	0.394	0.041*	0.337	0.037*	0.246	0.022*	0.847
Proteobacteria	0.156 ± 0.184	0.251 ± 0.127	0.015 ± 0.007	0.191 ± 0.107	0.310	0.041*	0.262	0.037*	0.285	0.161	0.660
Tenericutes	0.028 ± 0.038	0.009 ± 0.007	0.003 ± 0.002	0.087 ± 0.121	0.937	0.485	0.873	0.423	0.565	0.244	0.287
Verrucomicrobia	0.002 ± 0.002	0.010 ± 0.005	0.037 ± 0.035	0.003 ± 0.002	0.485	0.818	0.423	0.749	0.306	0.321	0.129

*^a^P-value of the two-group Mann–Whitney U-test. ^b^P-value of the Kruskal–Wallis rank sum test. ^c^P-value of the two-way ANOVA with a model including the interaction between host and habitat effects. ^∗^P < 0.05.*

### Compositional and Distributional Patterns of Bacteria across Hosts and Habitats

**FIGURE 2:**
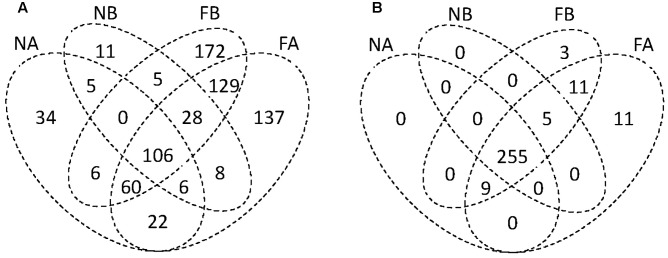
Venn diagrams showing the compositional similarity and uniqueness of **(A)** bacterial OTUs and **(B)** functional groups among four metacommunities. A, *Fejervarya limnocharis*; B, *Babina adenopleura*; F, farmland; N, forest.

**FIGURE 3:**
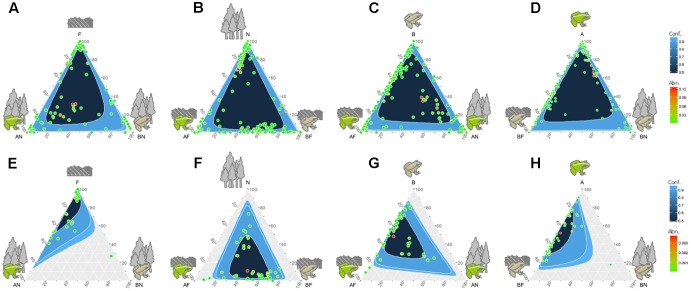
Ternary diagrams comparing the abundance (Abn) of gut bacterial composition **(A)** between different host frogs in the forest; **(B)** between different host frogs species in the farmland; **(C)** between the different habitats of *Fejervarya limnocharis*; **(D)** between the different habitats of *Babina adenopleura*; comparisons of the abundance of functional groups **(E)** between different host frogs in the forest; **(F)** bacteria between different host frogs in the farmland; **(G)** between the different habitats of *Fejervarya limnocharis*; **(H)** between the different habitats of *Babina adenopleura*. A, *Fejervarya limnocharis*; B, *Babina adenopleura*; N, forest; F, farmland.

Venn diagrams comparing all four sampling groups of gut microbiota (i.e., the gut microbial communities in two species in the farmland and in the forest sites) showed that 106 OTUs (14.54% of the total number) were found in all four groups (**Figure 2A**). There was a relatively high abundance of common gut microbes between the hosts in farmland sites (47.57%) as well as a higher RA than that found in forest sites (40.21%). On the other hand, *F. limnocharis* within different habitats shared more common gut microbes (35.86%) than *B. adenopleura* (25.93%, **Figure 2A**). Using ternary diagrams, we compared the bacterial distribution between hosts and between habitats (**Figures 3A–D**). In forest sites, we found most microbes common to both frog species were also common in farmland frogs (red points in **Figure 3A**). Forest-specific bacteria were also mostly host specific (i.e., clustering in the two corners of the triangle; **Figure 3A**). In contrast, when we compared the gut bacterial composition between hosts in farmland sites, we observed that the most dominant bacteria were in *F. limnocharis* although they were similar to forest frogs (red points in **Figure 3B**). Most farmland-specific bacteria were shared between the two hosts (i.e., located in the center of the bottom line; **Figure 3B**). This indicated that the gut microbes in forest sites were more divergent between frog species than those found in farmland sites. When we compared the bacterial distribution between habitats in both hosts separately, we saw similar patterns: very few of the common gut bacteria shared between habitats were found in both frog species. In the ternary diagram, the distribution was mainly concentrated around both sides and adjacent to the triangular vertex, in particular in the *B. adenopleura* (**Figures 3C,D**), which indicated the habitat divergence of gut bacteria. This inference was also confirmed by the significant difference for microbial RA between habitats based on random grouping (the Bray–Curtis similarity statistic *R* = 0.246, *P* = 0.013), although there was a non-significant difference between host species (*R* = -0.048, *P* = 0.736).

### Identifying Host and Habitat Specialists

**FIGURE 4:**
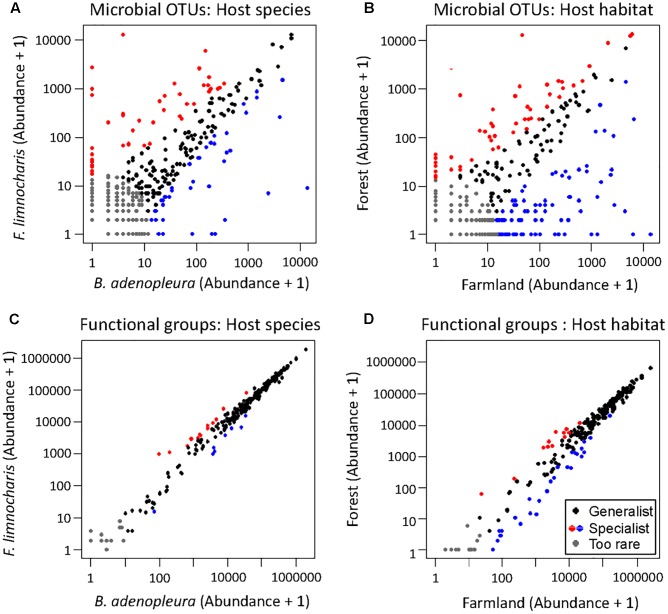
CLAM plots showing the grouping of generalists, specialists, and “too rare” bacteria **(A,B)**, and the functional groups **(C,D)** within gut microbial communities for different host species **(A,C)** and different host habitats **(B,D)**. These plots show that the microbial composition and the functional groups of the gut microbiota are more sensitive to habitat than the host. These gut bacteria reveal a highly functional convergence of the species assemblage.

**FIGURE 5:**
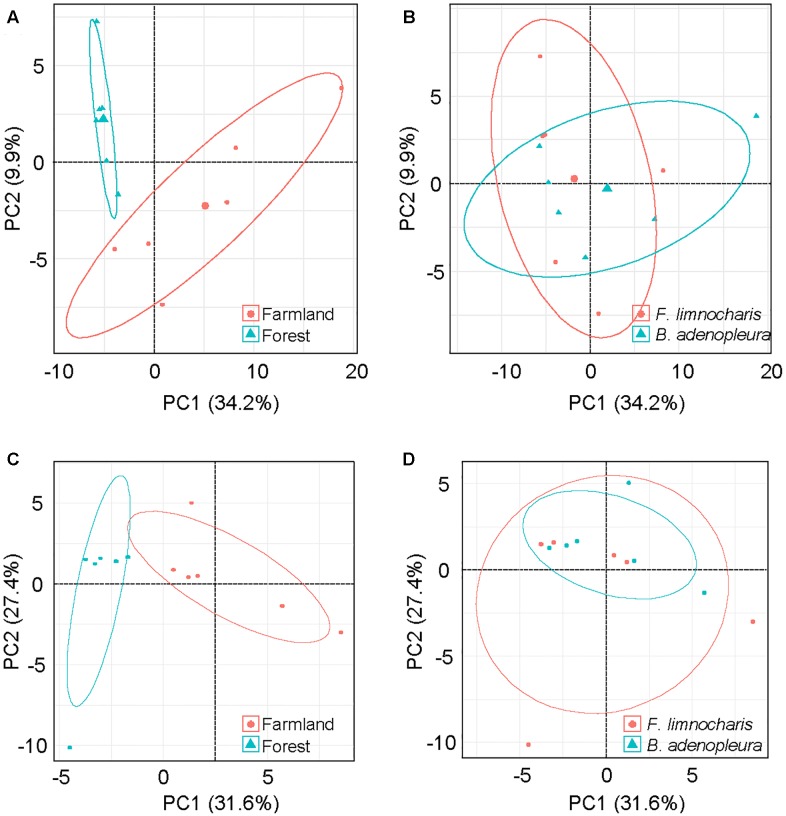
Principal component analysis (PCA) of gut microbial communities based on the relative abundance of **(A,B)** microbial phyla and **(C,D)** functional groups of gut bacteria. Samples from the microbial community were grouped by **(A,C)** habitat, and **(B,D)** host.

The top 3 common microbial phylum were used to verify the major composition between samples. No group can be clearly distinguished from other samples (Supplementary Figure S4). As the common microbes (i.e., the generalist or core microbiota) could confound estimates of habitat and/or host effects on gut microbial composition, we removed OTUs common to all habitats and hosts as well as those OTUs deemed too rare to be classified using the supermajority rule for assessing the host and habitat effects on gut microbial composition. By comparing different host species, we found 128 (17.6%) OTUs were generalists, 45 (6.2%) were specialists of *F. limnocharis*, 43 (5.9%) were specialists of *B. adenopleura*, and 513 (70.4%) were deemed “too rare” OTUs. When comparing habitats, 77 (10.6%) OTUs were generalists, 49 (6.7%) were specialists of *F. limnocharis*, 93 (12.8%) were specialists of *B. adenopleura*, and 510 (70.0%) were “too rare”-type OTUs (**Figure 4**). When comparing one group with the remaining samples, CLAM revealed similar results. There were 71.6–77.7% OTUs assigned to be “too rare,” and 4.5–11.5% OTUs were generalists to specific habitat of different host (Supplementary Figure S5). We retained the specialists of these two host/habitat datasets (157 OTUs, 21.5%) for subsequent analyses. First, we used PCA to verify the compositional differences of these specialists. Gut microbial communities from different habitats were clearly separated along in the first two principal components (explaining 44.1% of total variation, **Figure 5A**). However, the microbial communities were not distinguished between host species (**Figure 5B**). Similar results were also revealed in PERMANOVA showing that the variance of microbial community composition could be significantly explained by habitat effect (*R*^2^ = 0.379, *P* = 0.001) but not by host effect (*R*^2^ = 0.123, *P* = 0.109) nor by the joint effect of habitat × host (*R*^2^ = 0.084, *P* = 0.210, **Table 3**).

**Table 3:** Permutational multivariate analysis of variance (PERMANOVA) for testing the effect of habitat and host classification on the variance of gut bacterial composition and the functional groups of gut bacteria as estimated by the specialists of host species and host habitats.

	Gut microbial composition						Functional groups of gut bacteria				
	*df*	Sums of Sqs	Mean Sqs	F. model	*R*^2^	*P*	Sums of Sqs	Mean Sqs	F. Model	*R*^2^	*P*
Habitat	1	347.27	347.27	7.331	0.379	0.001^∗^	129.03	129.03	4.193	0.312	0.004^∗^
Host	1	112.67	112.67	2.379	0.123	0.109	25.95	25.95	0.843	0.063	0.521
Habitat × host	1	76.69	76.69	1.619	0.084	0.21	11.77	11.77	0.383	0.029	0.927
Residuals	8	378.98	47.37		0.414		246.16	30.77		0.596	
Total	11	915.61			1		412.92			1	

*^∗^P < 0.05. Sqs, sum of square.*

### Quantifying the Explanatory Variance of Gut Microbial Beta-Diversity Due to Habitat

Given that most analyses showed that the habitat was most responsible for determining the beta-diversity of gut microbiota, we then wished to examine the proportion of variance explained by the habitats. As such, we applied a dbRDA based on the Euclidian distance of RA for OTUs. The dbRDA showed that the constrained variables (habitat, frog species, and host × habitat) explained 31.3% of the variance (14.1, 8.0, and 9.3%, respectively), while unconstrained factors (68.7%) explained a larger proportion of variation. A further significance test via a type-II ANOVA on the effect of these two constraining factors showed that only habitat could significantly explain the beta-diversity of the gut microbiota (*P* = 0.004). Neither the host (*P* = 0.626) nor the host × habitat effects (*P* = 0.246; **Table 4**) explained gut beta-diversity. Thus, difference in habitat, despite representing only approximately a seventh of the total proportion of the variance (14.1%), governs the beta-diversity of frog gut microbial communities.

**Table 4:** Distance-based redundancy analysis for quantifying the significance of habitat and host effects on the gut bacterial composition and the functional groups of gut bacteria as estimated by the specialists of host species and host habitats.

	Gut microbial composition				Functional groups of gut bacteria			
	Sum of Sqs	Proportion	*F*	*P*	Sum of Sqs	Proportion	*F*	*P*
Total	1.33	1			8.337E-5	1		
Constrained	0.417	0.313			3.777E-5	0.453		
Habitat	0.187	0.141	1.624	0.004^∗^	2.165E-5	0.260	3.798	0.004^∗^
Host	0.106	0.080	0.922	0.626	1.217E-5	0.146	2.135	0.062
Habitat × host	0.123	0.093	1.08	0.246	3.951E-6	0.047	0.693	0.671
Unconstrained	0.913	0.687			4.560E-5	0.547		

*^∗^P < 0.05. Sqs, sum of square.*

### Prediction of Functional Content of Gut Bacteria Using the 16S rRNA Metagenome

As statistical analyses demonstrated that gut microbiota was differentiated by habitat, we investigated whether these gut bacteria function differentially on metabolism or physiology of host species. Bioinformatic functional profiling by PICRUSt (Langille et al., 2013) predicted 294 functional groups in the third-level KEGG pathways. Among them, we predicted 255 functional groups in all four sampling sets (**Figure 2B**) and 11 and 3 unique functional groups in *F. limnocharis* and *B. adenopleura*, respectively, in the farmland sites. Eleven functional groups were shared among frog species in the farmland sites (**Figure 2B**). In contrast, no unique or specific functional groups of gut bacteria were predicted for frogs from forest sites. The relatively high number of common functional groups (in contrast to the taxonomic composition of the gut microbiota) indicated highly conserved functions of gut microbiota among hosts and habitats (i.e., core functions). The farmland-specific functional groups of gut microbiota implied a higher niche differentiation between Anura species than within the highly disturbed habitats.

Such inference was supported by the ternary diagrams (**Figures 3E–H**). When we compared the two hosts in forest sites, most functional groups of gut bacteria were close to the vertex of the triangle (**Figure 3E**), indicating that there were no forest-specific functional groups. In contrast, the most abundant functional group, as well as most functions, were closer to the bottom line in **Figure 3F**, indicating a higher functional diversity in gut bacteria for the farmland frogs. This suggested that most metabolic or physiological functions were shared among the two frog species. When comparing functions between the different habitats in the two hosts, we observed a similar trend of more functional groups skewed toward the forest in both *F. limnocharis* (**Figure 3G**) and *B. adenopleura* (**Figure 3H**). This pattern argues for a higher functional diversity of frogs’ gut bacteria in farmland sites relative to forest sites.

### Identifying the Specific Functional Groups of the Gut Bacteria

**FIGURE 6:**
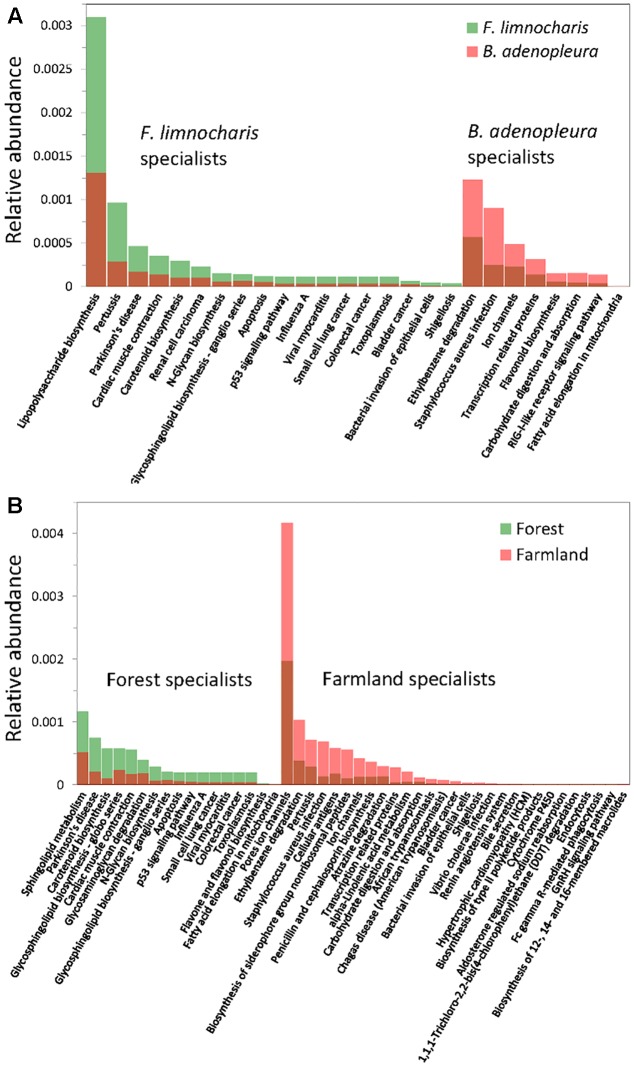
Relative abundance of the functional group specialists of gut microbiota. **(A)** Specialists of hosts; **(B)** specialists of habitats.

By supermajority rule, we identified 17 (5.78%) forest-specific and 29 (9.86%) farmland-specific functional groups of gut bacteria (**Figure 4D**). In addition, we identified 18 (6.12%) and 8 (2.72%) *F. limnocharis*-specific and *B. adenopleura*-specific functional groups, respectively (**Figure 4C**). Bar-plots (**Figure 6**) clearly highlight the differences in RA of these predicted functions for gut microbiota. We then used these specific functional groups to perform PCA, PERMANOVA, and dbRDA (as performed for accessing gut bacterial composition). Our results showed that (1) in PCA, the functional groups were grouped by host habitats (**Figure 5C**), although undistinguished by host species (**Figure 5D**); (2) PERMANOVA demonstrated that the habitat significantly explained the variance of functional groups (*P* = 0.004) but that neither hosts (*P* = 0.521) nor host × habitat (*P* = 0.927; **Table 3**) could account for the variance; and (3) the constraining factors explained 45.3% of the variance of functional groups of gut bacteria, in which the habitat, host, and host × habitat explained 26.0, 14.6, and 4.7% of the total variance, respectively. Only the habitat was able to significantly explain the beta-diversity of gut bacterial functions (*P* = 0.004; **Table 4**). An observed difference for the estimates of microbial composition is that the hosts had a marginally significant explanation for the functional divergence of gut bacteria (*P* = 0.062), revealing different physiological adaptabilities between the two frog species. These results (1) indicate a higher proportion of generalists in functional groups than in the overall gut bacterial composition, regardless of hosts or habitats; and (2) suggest that habitat remains the main factor governing the beta-diversity of the metabolic and physiological functions of frog gut microbiota.

## Discussion

Composition of the gut microbiota is a consequence of competition between the foreign and the native (resident) microbiota, which prohibit the establishment of both probiotic and pathogenic microbes (Lozupone et al., 2012). Experiments involving antibiotic treatments with Lewis rates indicate that exogenous interference reduced, rather than facilitated, the colonization by exotic bacteria, while the resident bacteria may be more plastic than previously thought (Manichanh et al., 2010). In mice, the intestinal microflora is affected by host genotypes, emphasizing the dependence of gut microbiota on a particular host (Deloris Alexander et al., 2006). If such phenomena are prevalent in animals, we hypothesized that the composition of gut microbiota should be governed by the endogenous gut environment that is shaped by the physical, physiological, and immune properties of host species, and would be less influenced by the surrounding environment. Our hypothesis is supported given the high proportion of common gut microbes (i.e., generalists, **Figures 4A,B**), and the most abundant common phylum as indication in other vertebrates (Ley et al., 2008), Bacteroidetes, Firmicutes, and Proteobacteria, do not show obvious differences in composition among habitat or host species (Supplementary Figure S4). This implies that the endogenous environment selects microbes that are optimally fit for the gastrointestinal characteristics. Nevertheless, a significant effect of habitat was detected by multiple statistical assessments (**Tables 3**, **4**). This effect was noted even given the large proportion of generalists (Supplementary Tables S3, S4, estimated by total 16S rRNA sequences) meaning that the external environments still positively affect the composition of gut microbiota (see, for example, Ley et al., 2006; Sullam et al., 2012; Wong and Rawls, 2012; Giatsis et al., 2015; Bletz et al., 2016; Chang et al., 2016).

**FIGURE 7:**
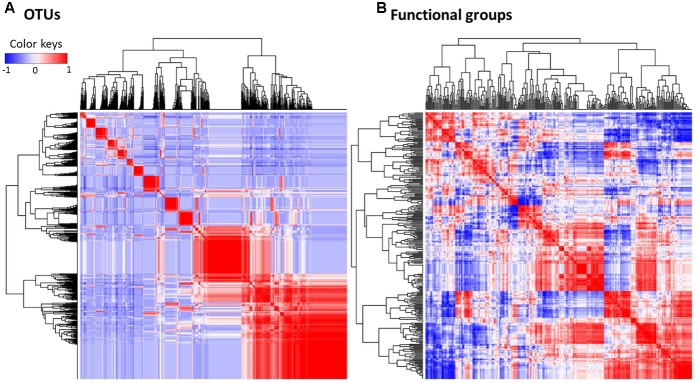
Spearman’s correlation of the relative abundance profiles, which were calculated between **(A)** one OTU and all other OTUs, or between **(B)** one predicted functional groups and other groups of the gut bacteria. The tighter and more distinguishable clusters in bacterial OTUs **(A)** in contrast to the more dispersed and indistinguishable clusters of the functional groups **(B)** suggest more stable physiological functions compared to the composition of the gut microbial assemblage in changing environments. Red and blue colors indicate the positive and negative correlations, respectively. Color intensity represents the strength of correlation.

However, changes in the composition of the gut microbiota as the habitat is altered might not severely impact core physiological functions of gut microbiota. In metabolic functional predictions, a relatively higher proportion of functional generalists was inferred than generalists for microbial OTUs, implying that most of the replacing gut bacteria still harbored similar physiological functions (e.g., functional redundancy). This pattern could be beneficial for increasing the resilience and persistence of the functional stability of gut microbiota (Elmqvist et al., 2003; Lozupone et al., 2012). In other words, the stable physiological states in a changing gut environment are preserved (Bletz et al., 2016) by a tight interaction between microbial metabolic activities rather than the taxonomic composition of microbes. This is also shown by a more significant correlation, despite a greater dispersal, on a heat map of the microbial metabolic functional groups than of the microbial composition (**Figure 7** and Supplementary Table S5). Such greater significant correlations in functional cohorts compared with taxonomic cohorts implies physiological links rather than phylogenetic associations between co-occurring microbes in frog gut microbiota.

The environment in which the host lives affects gut microbial diversity (Giatsis et al., 2015; Bletz et al., 2016; Chang et al., 2016). Such influence can be mediated by environment–diet–microbe–host interactions (Zhernakova et al., 2016). Natural habitats (e.g., forest) are believed to have diversified nutrient and food resources because of the presence of a healthier ecosystem (Polis et al., 1997) that harbors more diverse (gut) microbiota (Amato et al., 2013; Chang et al., 2016). A global study demonstrated a positive correlation between the plant community and environmental microbial community (Leff et al., 2015), confirming the positive impact of ecosystem diversity on microbial diversity. In our study, however, the farmland frogs, which are subject to frequent environmental disturbance due to agricultural activities, have a higher gut bacterial diversity and more habitat-specific gut bacteria than forest frogs (**Figures 2A**, **4B**). This result is contrary to previous studies suggesting a positive association between the ecosystem health and gut microbial diversity inferred in primates (Amato et al., 2013; Barelli et al., 2015). Surprisingly, farmland frogs harbored a more diversified gut bacterial flora than the forest frogs, in particular species-specific microbes (**Figure 2A**). The ternary diagram also showed an obvious skew toward the farmland frogs in terms of functional groups (**Figures 3G,H**). Under a disturbed environment, frog species should undergo some stress such as changes in food resources (Chang et al., 2016) that may alter the physiological conditions of frogs and further influence the gut environment and impact on the gut microbiota (Toft and Andersson, 2010). Our previous study inferred a high risk of invasive disease in frogs in farmlands due to a relatively high proportion of the phyla Proteobacteria, Actinobacteria, Acidobacteria, and Planctomycetes in frog guts (Chang et al., 2016). We suggest that such highly diversified gut microbiota of farmland frogs reflects their physiological, metabolic, and ecological responses to environmental disturbance. More specialized functional groups that skew toward gut bacteria in the farmland habitat (**Figures 3E–H**) also support a hypothesis of high “functional response diversity” of the gut microbiota in farmland frogs to compensate for the environmental disturbance (Elmqvist et al., 2003).

Although the physiological and metabolic functions of the gut microbiota are more stable than the microbial composition between different environments (**Figure 4**; Bletz et al., 2016), specialized functional groups seem more capable of responding to given habitat characteristics. Among them, the functional specialists of forest frog microbiota are mostly the physiological and cellular metabolites, while those of farmland frog microbiota are composed additionally of functions related to pesticide degradation (ethylbenzene, atrazine, and DDT degradation) and pathogenic diseases (e.g., pertussis, *Staphylococcus aureus* infection, penicillin and cephalosporin biosynthesis, trypanosomiasis, bacterial invasion of epithelial cells, Shigellosis, and *Vibrio cholerae* infection) (**Figure 6B**). Since the microbial community is the unit of selection under specific conditions (Day et al., 2011), properties of microbial composition and the ecological functions of these microbial communities could be seen as the elements that reflect the hosts’ survival risks (Weng et al., 2016). This inference of increased pathogenic bacteria and changes of physiological functions under artificial interference on amphibian gut microbiota is similar to the consequence of the increasing risk of bacterial infections under hibernation induced in laboratory (Weng et al., 2016). Given these results, gut microbial ecosystem not only mirrors the ecological condition of the habitat, but also reflects the fitness (e.g., health) of host species in that environment. Hence, our results also indicate that, even if an amphibian may not be sensitive to environmental pollution (Kerby et al., 2010), its gut microbes are sensitive.

Such functional specialists were also illustrated between hosts (**Figure 6A**). Functional groups related to the health status were highly abundant in *F. limnocharis* (e.g., pathogenic diseases: pertussis, influenza A, viral myocarditis, toxoplasmosis, Shigellosis, bacterial invasion of epithelial cells; physical health: cardiac muscle contraction, cancers; **Figure 6A**). We also found an interesting link to several cancer-related functions (renal cell carcinoma, small cell lung cancer, colorectal cancer, bladder cancer, N-glycosylation, glycosphingolipid biosynthesis, apoptosis, p53 signaling pathway) for the gut microbiota of *F. limnocharis* (**Figure 6A**). Several studies have also shown links between cancer and the gastrointestinal microbiome (Schwabe and Jobin, 2013; Bultman, 2014; Hullar et al., 2014; Johnson et al., 2016). The gut microbiota may reciprocally affect, and be affected by, the mucosal integrity, development, and activity of immune system of hosts (Schwabe and Jobin, 2013), reflecting the health status of hosts. Abundant disease-related functions in the gut microbiota of *F. limnocharis* imply the poor adaptation of this species within our sampling area, or under strong selective pressure of disease-associated inflammation (Börnigen et al., 2013; Kreisinger et al., 2014; Loudon et al., 2014). In contrast, *B. adenopleura* harbors a greater number of functions related to pesticide degradation (e.g., ethylbenzene degradation; **Figure 6A**). The uses of pesticide and agro-chemical were common in amphibian habitat, especially for frog inhabit near the farmland of Northern Taiwan, which is corresponding to our sampling sites (Lin et al., 2008), indicating that the selective pressure in farmland (e.g., pesticide and pathogens) is asymmetric for the two different frog species, despite there are still several factors which may be different among our sampling sites (e.g., Temperature or elevation) which may also contribute to the gut microbial composition difference between habitats. Therefore, we may not confidently conclude that differences in gut microbial compositions between samples were due to uses of pesticide. Our study suggests that compositional and functional prediction of gut microbiota reflects the specific environmental adaptability of adult frogs (Toft and Andersson, 2010).

## Author Contributions

P-CL and C-WC conceived and designed the experiments. C-WC contributed frog materials. B-HH performed the laboratory experiments. B-HH, C-WH, JG, and P-CL analyzed the data. P-CL wrote the paper. B-HH, C-WC, JG, and C-WH critically reviewed the manuscript. All authors participated in the discussion and read and approved the final manuscript.

## Conflict of Interest Statement

The authors declare that the research was conducted in the absence of any commercial or financial relationships that could be construed as a potential conflict of interest.
