# Supplementary material for: Composition and Functional Specialists of the Gut Microbiota of Frogs Reflect Habitat Differences and Agricultural Activity
Source: Front Microbiol. 2018 Jan 11;8:2670. doi: 10.3389/fmicb.2017.02670 (PMC5768659; doi:10.3389/fmicb.2017.02670)
Supplement: Supplementary file 1 [file Table_1.PDF]

Supplementary Table

Composition and functional specialists of the gut microbiota of frogs reflect habitat differences and agricultural pesticide pollution

Bing-Hong Huang[1], Chun-Wen Chang[1,2], Chih-Wei Huang[1], Pei-Chun Liao[1]

Supplementary Table S1 Habitat preferences of *Babina adenopleura* and *Fejervarya limnocharis*.

Species	Habitats		Reference
	Tadpole	Adult	
Babina adenopleura	Bottom dweller	-	(Xu et al 2004)
	-	Ponds, streams, and marshes	(Hsu et al 2005)
	-	Ponds and forest	(Shih et al 2006)
	-	Ponds, lake, and slowly flowing streams	(Chuang 2006)
	-	Grasslands and ponds	(Jung 2011)
	-	Permanent ponds and less protected areas	(Lu and Chen 2012)
	-	Quiet, shallow, small water bodies having leaf litter accumulation, with sandy and poorly vegetated shores	(Goutte et al 2013)
Fejervarya limnocharis	Bottom dweller	-	(Heyer 1973)
	-	Ponds and marshes	(Hsu et al 2005)
	-	Agricultural lands, reclaimed lands, and ponds	(Shih et al 2006)
	-	Densely vegetated areas, shallow flowing water zones	(Lee et al 2006)
	-	Agricultural lands and grasslands	(Jung 2011)
	-	Shallow waterbodies and rice fields	(Ma 2012)
