# Supplementary material for: Composition and Functional Specialists of the Gut Microbiota of Frogs Reflect Habitat Differences and Agricultural Activity
Source: Front Microbiol. 2018 Jan 11;8:2670. doi: 10.3389/fmicb.2017.02670 (PMC5768659; doi:10.3389/fmicb.2017.02670)
Supplement: Supplementary file 2 [file Table_2.PDF]

Supplementary Table S2 Diets of *Babina adenopleura* and *Fejervarya limnocharis*

Species	Diet	Reference
Babina adenopleura	Arachnida, Coleoptera, Hemiptera, Hymenoptera, Isoptera, Lepidoptera, Orthoptera, Stylommatophora	(Wang 2013)
Fejervarya limnocharis	Arachnida, Blattodea, Coleoptera, Collembola, Dermaptera, Diptera, Hemiptera, Hemiptera, Hymenoptera, Isoptera, Orthoptera, Stylommatophora	(Ma et al 1992)
	Coleoptera, Hemiptera, Hymenoptera, Lepidoptera, Orthoptera	(Zhou et al 1996)
	Arachnida, Hymenoptera, Lepidoptera	(Jiang 2013)
	Aranea, Coleoptera, Collembola, Dermaptera, Diptera, Hemiptera, Hymenoptera, Isoptera, Lepidoptera, Orthoptera, Psocoptera	(Norval et al 2014)
