# Supplementary material for: Composition and Functional Specialists of the Gut Microbiota of Frogs Reflect Habitat Differences and Agricultural Activity
Source: Front Microbiol. 2018 Jan 11;8:2670. doi: 10.3389/fmicb.2017.02670 (PMC5768659; doi:10.3389/fmicb.2017.02670)
Supplement: Supplementary file 3 [file Table_3.PDF]

Supplementary Table S3 Permutational multivariate analysis of variance (PERMANOVA) for testing the effect of the habitat and host classification on the variance of gut bacterial composition and the functional groups of gut bacteria as estimated with total 16SrRNA sequencing data

		Gut microbial composition					Functional groups of gut bacteria				
	df	Sums of Sqs	Mean Sqs	F.Model	R^2	*P*	Sums of Sqs	Mean Sqs	F.Model	R^2	*P*
Habitat	1	981.4	981.45	2.563	0.204	0.006*	579.02	579.02	3.443	0.257	0.003*
Host	1	369.5	369.51	0.965	0.077	0.477	171.41	171.41	1.019	0.076	0.335
Habitat×Host	1	399.5	399.47	1.043	0.083	0.381	152.93	152.93	0.909	0.068	0.436
Residuals	8	3063.4	382.93		0.636		1345.48	168.19		0.598	
Total	11	4813.9			1		2248.85			1	
