# Supplementary material for: Composition and Functional Specialists of the Gut Microbiota of Frogs Reflect Habitat Differences and Agricultural Activity
Source: Front Microbiol. 2018 Jan 11;8:2670. doi: 10.3389/fmicb.2017.02670 (PMC5768659; doi:10.3389/fmicb.2017.02670)
Supplement: Supplementary file 4 [file Table_4.PDF]

Supplementary Table S4 Distance-based redundancy analysis for quantifying the significance of habitat and host effects on the gut bacterial composition and the functional groups of gut bacteria as estimated with total 16SrRNA sequencing data

	Gut microbial composition				Functional groups of gut bacteria			
	Sum of Sqs	Proportion	*F*	*P*	Sum of Sqs	Proportion	*F*	*P*
Total	1.380	1			4.83E-03	1		
Constrained	0.440	0.319			1.79E-03	0.371		
Habitat	0.196	0.142	1.668	0.009*	8.17E-04	0.169	2.149	0.055
Host	0.107	0.078	0.911	0.651	5.08E-04	0.105	1.3365	0.207
Habitat×Host	0.136	0.099	1.161	0.137	4.66E-04	0.096	1.2262	0.262
Unconstrained	0.940	0.681			3.04E-03	0.629		
