# Supplementary material for: Composition and Functional Specialists of the Gut Microbiota of Frogs Reflect Habitat Differences and Agricultural Activity
Source: Front Microbiol. 2018 Jan 11;8:2670. doi: 10.3389/fmicb.2017.02670 (PMC5768659; doi:10.3389/fmicb.2017.02670)
Supplement: Supplementary file 6 [file Image_1.PDF]

Supplementary Figure

Composition and functional specialists of the gut microbiota of frogs reflect habitat differences and agricultural pesticide pollution

Bing-Hong Huang[1], Chun-Wen Chang[1,2], Chih-Wei Huang[1], Pei-Chun Liao[1]

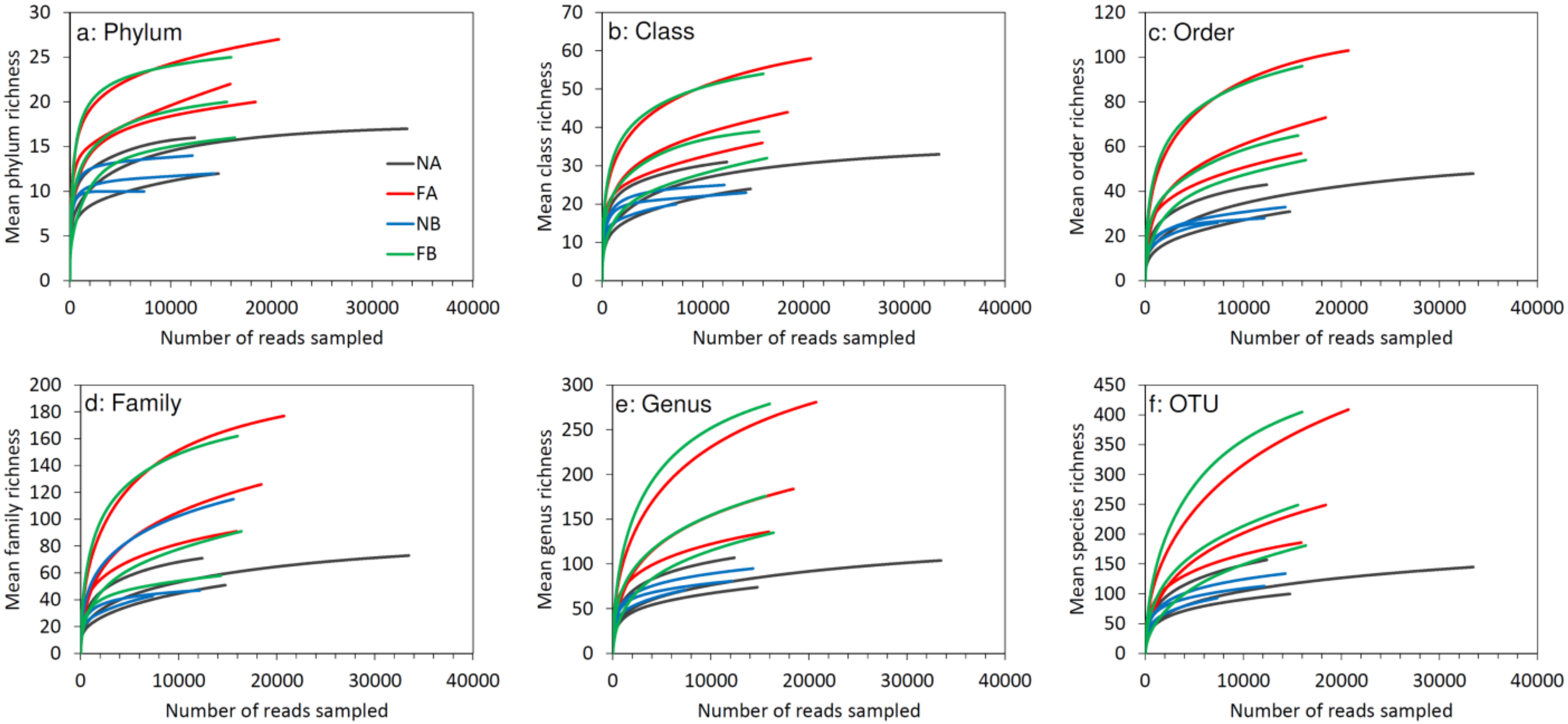

Supplementary Figure S1. Rarefaction curves for the intestinal microbial communities of *Fejervarya limnocharis* and *Babina adenopleura* in natural habitats (NA and NB, respectively) and in farmland (FA and FB, respectively) at different taxonomic levels: (A) phylum, (B) class, (C)

order, (D) family, (E) genus, and (F) OTU.

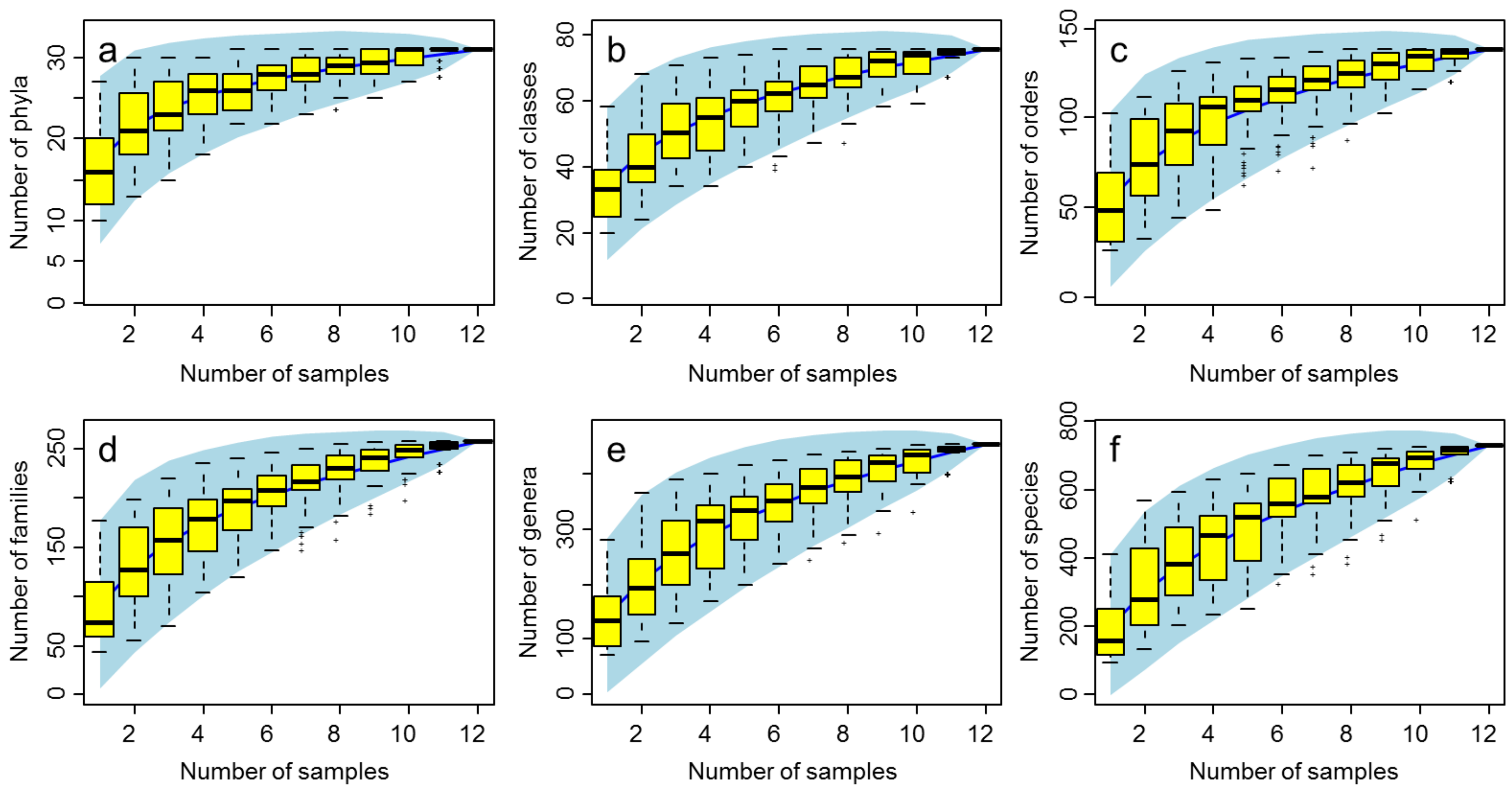

Supplementary Figure S2**.** Species accumulation curves (SAC) showing the relationship between the number of sampled host species and the richness of gut microbiota estimated for different taxonomic levels.

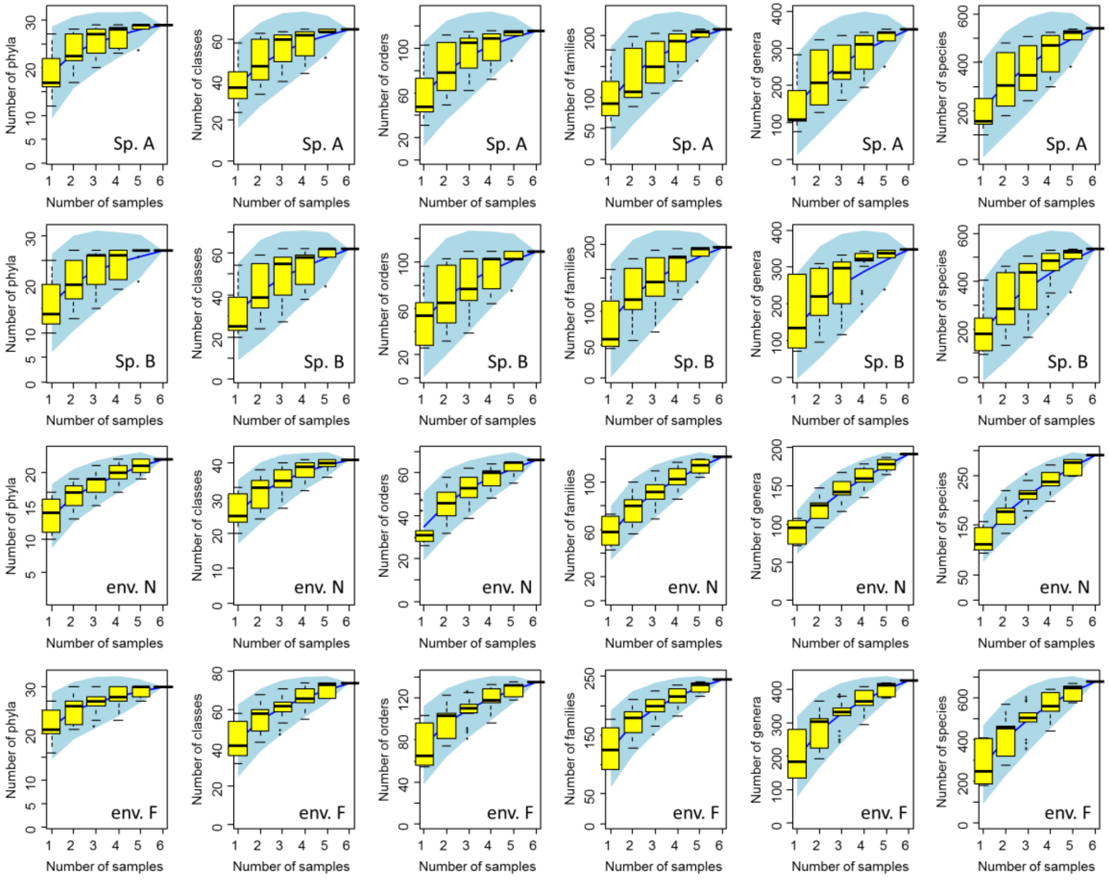

Supplementary Figure S3. Species accumulation curves (SAC) of four groups of gut microbial communities (metacommunities) estimated for different taxonomic levels. Sp. A: samples of *Fejervarya. limnocharis*; Sp. B: samples of *Babina adenopleura*; N: samples from natural habitat sites; F: samples from farmland sites.

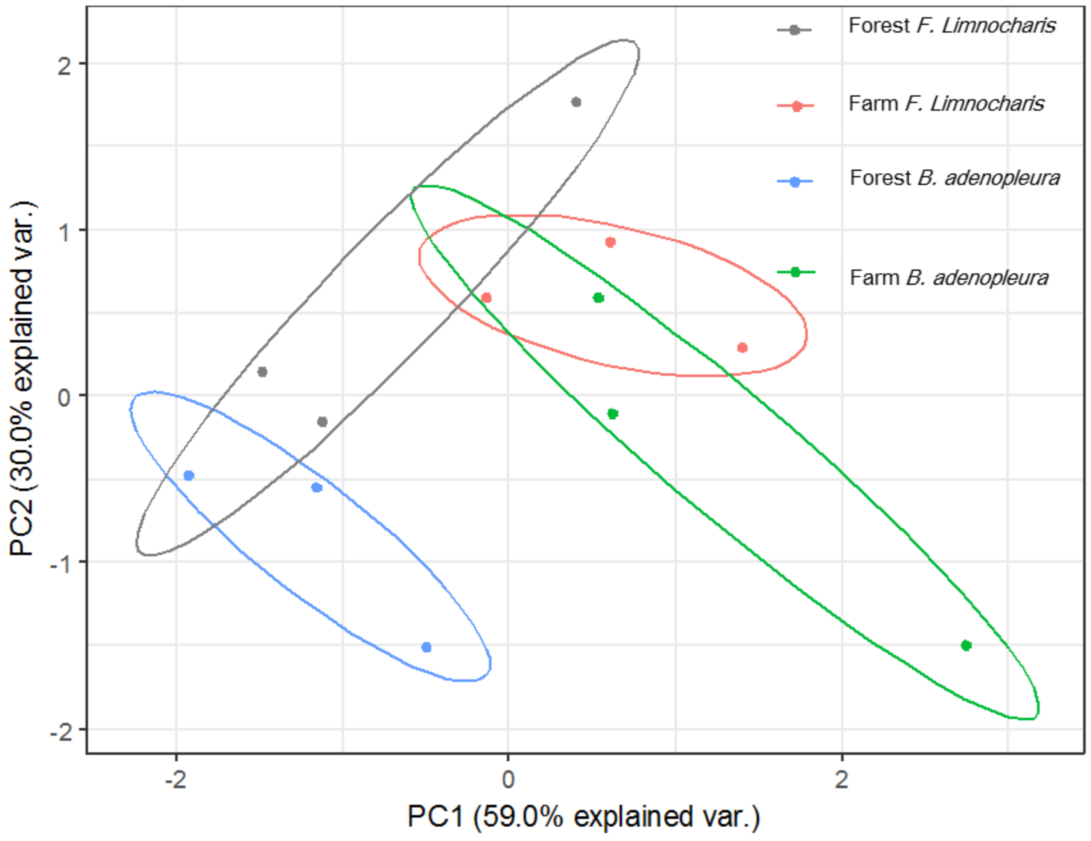

Supplementary Figure S4. Principal component analysis (PCA) of gut microbial communities based on the relative abundance of the top 3 abundant phylums.

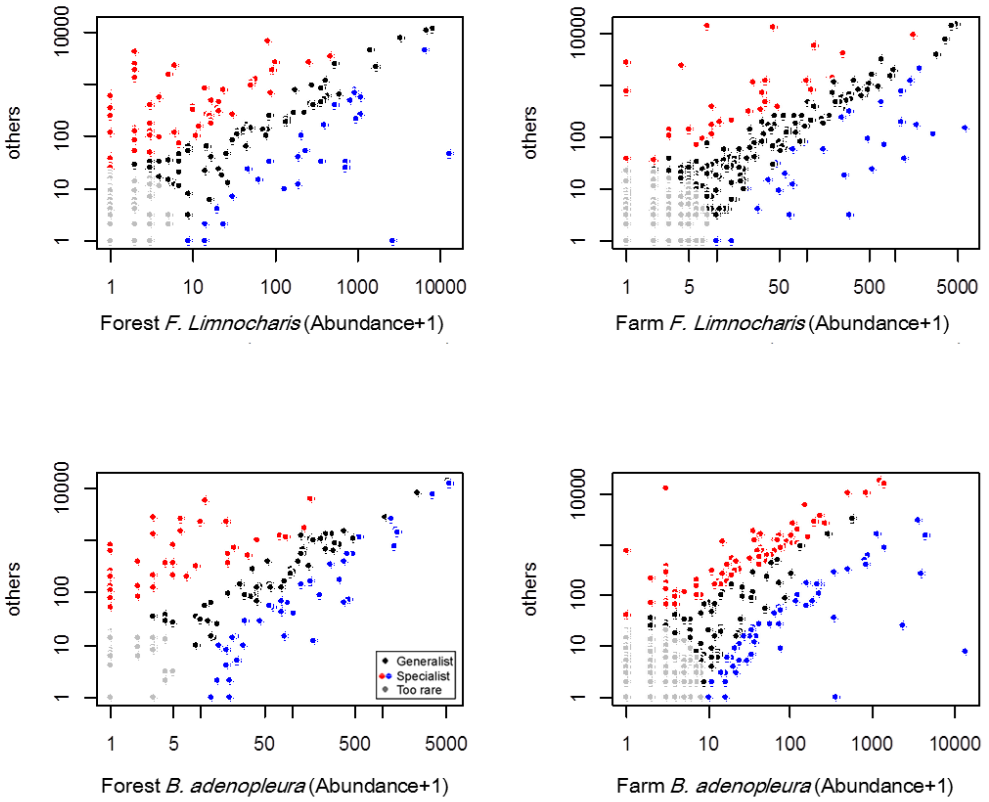

Supplementary Figure S5. CLAM plots showing the grouping of generalists, specialists, and "too rare" bacteria of different sampling groups comparing to the remaining samples.
